# Aortic Dimensions in a South Asian Population: Establishing Normative Data and Implications for Clinical Practice

**DOI:** 10.1055/a-2776-6270

**Published:** 2026-01-14

**Authors:** Bijoy G. Rajbanshi, Bhuwan Kayastha, Gangaram Biswakarma, Sangam KC, Pralaya Khadka, Dharmendra Joshi, Malakh L. Shrestha, Carlos A. Mestres, Ram Kumar Ghimire

**Affiliations:** 1 Department of Cardiovascular and Thoracic Surgery, Nepal Mediciti, Lalitpur, Nepal; 2 Department of Radiology, Nepal Mediciti, Lalitpur, Nepal; 3 Central Department of Management, Tribhuvan University, Kathmandu, Nepal; 4 Department of Cardiovascular Surgery, Mayo Clinic, Rochester, Minnesota, United States; 5 Department of Cardiothoracic Surgery and the Robert WM Frater Cardiovascular Research Center, The University of the Free State, Bloemfontein, South Africa

**Keywords:** aorta, size, South Asian

## Abstract

**Background:**

Diameter of the aorta is a significant contributor and predictor for complications and a fundamental parameter for intervention. It is recognized that age, sex, and ethnicity play a role in aortic size. We thus sought to determine the normal dimensions among our population.

**Methods:**

A retrospective analysis of images was done of all polytrauma patients admitted between January 2018 and December 2022 who underwent protocolized noncontrast computed tomography of the chest and abdomen to measure the aortic diameter at established reference points.

**Results:**

There were 513 patients; the mean age was 36.5 ± 14.6 (range: 18–86), and 382 (74.5%) were males. Aortic dimensions at sinus, mid-ascending, arch, descending, suprarenal, and infrarenal aorta were 30.7 ± 3.8, 29.3 ± 4.5, 24.9 ± 3.3, 20.1 ± 3.0, 19.4 ± 2.9, and 15.3 ± 2.2, respectively. Age demonstrated a positive correlation to the diameter at the ascending, descending, and infrarenal aorta ( *r* = 0.58, *p* = 0.001 [95% confidence interval, CI = 0.519; 0.634]; *r* = 0.69, *p* = 0.001 [95% CI = 0.641; 0.732]; *r* = 0.57, *p* = 0.001 [95% CI = 0.509; 0.626]) along with the length of the ascending aorta ( *r* = 0.420, *p* = 0.001 [95% CI = 0.345; 0.488]; *r* = 0.536, *p* = 0.001 [95% CI = 0.471; 0.595]; *r* = 0.476, *p* = 0.001 [95% CI = 0.406; 0.540), respectively. There was a positive correlation of aortic diameters to body mass index (BMI), systolic blood pressure (SBP), and diastolic blood pressure (DBP). Females had smaller dimension at the reference points, but without any statistical significance. There were 50 (9.8%) patients with bovine aortic arch and 10 (1.9%) with separate origin of left vertebral artery.

**Conclusion:**

Normal values of the diameter of the aorta for a South Asian population are provided. Diameter is affected by age, length of the ascending aorta, BMI, SBP, and DBP. This study suggests that the aorta size is smaller in the South Asian population than the referenced Western population, more significantly for distal descending and abdominal aorta and that ethnicity plays a role in determining aortic dimensions.

## Introduction

It is well recognized that the dimension of the aorta is a significant predictor of aortic complications. 1 2 3 Prevention of acute aortic syndrome is the primary goal of elective surgical intervention and is based on identifying those aortas at risk by virtue of their maximum diameter, which is determined by diagnostic imaging, and then by pursuing surgical intervention.

There is a lack of robust data on the normal size of the aorta in the general population; thus, the normal size of the aorta measured across various sites remains under discussion. Normal aorta size differs significantly across the population based on genetics, age, sex, body habitus, height, and ethnicity. 2 4 5 While selected research has been conducted on the aortic dimension in discrete populations in the West, 6 7 8 there remains a significant knowledge gap regarding the aortic dimensions in the Asian population.

Further, there remains ambiguity with regard to the most appropriate size for aortic intervention. Complications do occur at smaller sizes of the aorta than recommended for intervention, more so for the descending aorta (DAo) and abdominal aorta (ABAo) in comparison to the ascending aorta (AAo). 2 9 10 11 12 13 14

We sought to determine the size of the aorta at various reference points among our population in South Asia.

## Materials and Methods

### Study Design

This is a retrospective study of all patients who were admitted to our institution for polytrauma who have undergone a protocolized noncontrast computed tomography (CT) scan of the chest and abdomen for assessment of injuries.

### Inclusion/Exclusion Criteria

All patients with polytrauma who underwent a noncontrast CT scan of the chest and abdomen during the period from January 2018 to December 2022 were included. Patients who were below 18 years of age, individuals not confirming to Asian ethnicity, and patients with poor quality imaging for aortic assessment were excluded from the study.

### Imaging Protocol

Noncontrast CT study was performed with a 128-slice MDCT (Siemens SOMATOM Definition AS, Siemens Healthineers, Erlangen, Germany) with the following acquisition parameters: 120 kV, 135 mAs with slice/collimation of 128 × 0.6 mm with gantry rotation time of 0.33 seconds. Images were obtained from the level of skull base to mid-thigh, and 1.5-mm axial images were reconstructed. Images were evaluated in soft tissue windows in three multiplanar reformatted planes (axial, coronal, and sagittal). These images were used to assess the dimensions of the aorta at well-established reference points.

The diameters of the aorta were obtained by measuring the aorta at the level of the annulus, aortic sinuses, sinotubular junction, AAo at its widest diameter; mid aortic arch between left common carotid and left subclavian artery; DAo—just distal to the origin of left subclavian artery, distal DAo just above the aortic hiatus; proximal ABAo just proximal to the celiac trunk; and infrarenal ABAo just distal to the renal artery origin. The length of the AAo was measured in coronal reconstructed images from the sinotubular junction to the level at the origin of the brachiocephalic trunk identifying following a centerline.

Measurement of the aorta was obtained by centerline measurement following multiplanar reformatting and measuring at the widest diameter from the outer border to the outer border avoiding obliquity and remaining perpendicular to the long axis of the aorta and the axis of blood flow. Measurements at the root were done following double oblique CT images obtained from three-dimensional workstation and reconstructed in axial images perpendicular to the long axis of the aorta to avoid obliquity. All the images were reviewed by authors B.G.R. and B.K.

### Definitions

Hypertension was defined as systolic blood pressure (SBP) > 140 mm Hg, diastolic blood pressure (DBP) > 90 mm Hg, or self-reported hypertension with the use of antihypertension medications. The SBP and DBP were measured with an automated sphygmomanometer using a cuff of appropriate size.

South Asian race was defined as the Nepalese population, which comprises of a mixture of Caucasian and Oriental origin. Presently, the Nepalese ethnic diaspora accounts for a majority of Indo–Europeans (82.1%) with a smaller contribution by Sino–Tibetan–Burman descent (17.3%). 15

### Ethics

Informed consent was waived because the study is a retrospective review. The study was approved by the Ethical Review Board of the National Health Research Council, Kathmandu, Nepal (Reference Number: 1843-9/2020 P-February 23, 2020).

### Statistical Analysis

The descriptive statistics for categorical variables are reported as frequencies and percentages, continuous variables as mean ± standard deviation or median and ranges as appropriate. The correlation analysis for the continuous variables was analyzed with Pearson correlation coefficient ( *r* -value). Similarly, the association between the categorical variables was investigated using the chi-square (λ) value to assess for a relationship with aorta size at the sinus, AAo, distal DAo, and infrarenal aorta. The significance level was estimated with 95% confidence interval (CI) and *p* -values.

## Results

### Baseline Characteristics

Between January 2018 and December 2022, 704 patients were admitted and underwent CT scans of the chest and abdomen. Excluded were those below 18 years of age ( *N* = 50), individuals not confirming to Nepalese ethnicity ( *N* = 6), and patients with excessive motion artifact images resulting in difficulty in assessing the aortic diameters ( *N* = 135). A total of 513 patients were included in the study, with a mean age of 36.5 ± 14.6 years, a median age of 33 years (range: 18–86 years), and 382 (74.5%) were males. The demographics are listed in Table 1 . There were 50 (9.8%) patients with bovine aortic arch (predominant Type II) and 10 (1.9%) patients with a separate origin of vertebral artery from the arch of the aorta ( Table 1 ).

**Table 1:** Demographics

	Number	%
Age in years	36.5 ± 14.6	Median 33 (R18–86)
Gender—male	382	74.5
Bovine aortic arch	50	9.8
Bovine arch Type I	23	4.5
Bovine arch Type II	27	5.3
Vertebral artery	10	1.9
Polycystic kidney	1	0.2
Smoking	30	5.8
Family history of CAD	1	0.2
DM	44	8.6
Dyslipidemia	10	1.9
Hypertension	71	13.8
Radiation	1	0.2
Systolic BP	124.0 ± 16.8	
Diastolic BP	75.7 ± 10.2	

Abbreviations: BP, blood pressure; CAD, coronary artery disease; DM, diabetes mellitus.

**Fig. 1:**
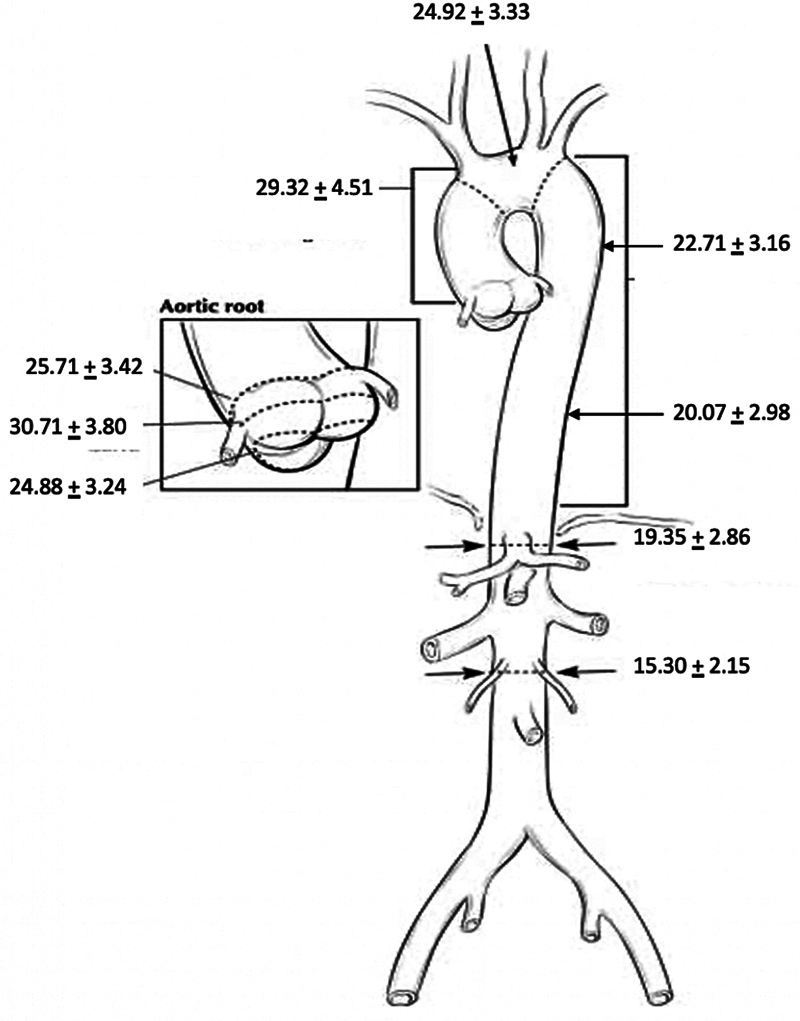
Normal aortic diameter. Reproduced with permission from Berger and Elefteriades. 26

The dimensions of the aorta at the level of the annulus, sinus, AAo, arch, proximal and distal DAo, suprarenal, and infrarenal ABAo were 24.9 ± 3.2, 30.7 ± 3.8, 29.3 ± 4.5, 24.9 ± 3.3, 22.7 ± 3.2, 20.1 ± 3.0, 19.4 ± 2.9, and 15.3 ± 2.2 mm, respectively ( Table 2 , Fig. 1 ).

**Table 2:** Aorta size, gender distribution, aorta size index

	Aorta size	Aorta size (male)	Aorta size (female)	Aorta size index
Aortic annulus	24.9 ± 3.2	25.4 ± 3.1	23.4 ± 3.2	14.5 ± 2.1
Aortic sinus	30.7 ± 3.8	31.4 ± 3.7	28.6 ± 3.3	18.0 ± 2.4
Sinotubular junction	25.7 ± 3.4	26.2 ± 3.4	24.3 ± 3.2	15.0 ± 2.2
Ascending aorta	29.3 ± 4.5	29.3 ± 4.5	29.3 ± 4.4	17.1 ± 2.9
Aortic arch	24.9 ± 3.3	24.9 ± 3.3	25.0 ± 3.5	14.6 ± 2.2
Proximal descending aorta	22.7 ± 3.2	23.0 ± 3.1	22.0 ± 3.3	13.3 ± 1.9
Distal descending aorta	20.1 ± 3.0	20.3 ± 3.0	19.4 ± 2.9	11.8 ± 1.8
Suprarenal abdominal aorta	19.4 ± 2.9	19.5 ± 2.8	18.8 ± 2.9	11.3 ± 1.8
Infrarenal abdominal aorta	15.3 ± 2.2	15.5 ± 2.1	14.7 ± 2.1	9.0 ± 1.3
Ascending aorta length	63.3 ± 8.7	64.0 ± 8.6	61.0 ± 8.6	

Note: Measurements in mm.

The size of the aorta in females was predominantly smaller than that of males, yet without any statistical significance ( Table 2 ). The aorta size index at the reference points is listed in Table 2 . The aorta size among various age groups is listed in Table 3 , and the aorta height index is listed in Table 4 .

**Table 3:** Aorta size based on age group

Age group in years (aorta size in mean ± SD)	18–30 ( *N* = 227)	31–40 ( *N* = 117)	41–50 ( *N* = 81)	51–60 ( *N* = 52)	61–70 ( *N* = 22)	>70 ( *N* = 14)
Ao annulus	24.4 ± 3.0	24.9 ± 3.2	25.7 ± 3.2	25.6 ± 3.9	25.1 ± 2.9	26.24 ± 4.48
Ao sinus	29.6 ± 3.5	30.9 ± 3.6	32.0 ± 3.5	32.1 ± 4.5	31.9 ± 4.3	31.89 ± 3.62
Ao STJ	24.6 ± 3.3	25.9 ± 2.9	26.8 ± 3.4	27.6 ± 3.5	26.9 ± 4.0	26.83 ± 2.32
Asc Ao	27.0 ± 4.2	28.9 ± 3.2	31.4 ± 3.1	33.6 ± 3.3	34.0 ± 4.2	33.91 ± 3.45
Ao arch	23.2 ± 2.9	24.8 ± 2.7	26.7 ± 2.7	27.5 ± 2.6	28.0 ± 3.3	29.02 ± 2.81
Proximal DAo	20.9 ± 2.4	22.6 ± 2.6	24.4 ± 2.7	25.6 ± 2.5	26.4 ± 1.7	26.69 ± 1.87
Distal DAo	18.3 ± 2.2	20.0 ± 2.2	21.6 ± 2.1	23.1 ± 2.4	23.3 ± 2.7	25.14 ± 2.35
Suprarenal Ao	17.5 ± 2.0	19.3 ± 2.1	20.8 ± 1.8	22.5 ± 2.5	22.8 ± 2.8	24.24 ± 1.40
Infrarenal Ao	14.2 ± 1.7	15.2 ± 1.7	16.2 ± 1.7	17.2 ± 2.4	17.1 ± 2.1	18.53 ± 1.79
Asc Ao length	59.7 ± 8.0	63.1 ± 7.8	66.5 ± 7.3	69.0 ± 8.1	68.5 ± 7.7	74.21 ± 8.08

Abbreviations: Ao, aorta; ASC, ascending; DAo, descending aorta; SD, standard deviation; STJ, sinotubular junction.

Note: Measurement in mm.

**Table 4:** Aorta length index

Aorta height index ( *N* = 337)	Mean ± standard deviation
Ao annulus	14.5 ± 2.1
Ao sinus	18.0 ± 2.4
Ao STJ	15.0 ± 2.2
Asc Ao	17.1 ± 2.9
Ao arch	14.6 ± 2.2
Proximal DAo	13.3 ± 1.9
Distal DAo	11.8 ± 1.8
Suprarenal Ao	11.3 ± 1.8
Infrarenal Ao	9.0 ± 1.3

Abbreviations: Ao, aorta; Asc, ascending; DAo, descending aorta; STJ, sinotubular junction.

We assessed for correlation or association of different variables that could affect the size of the aorta at the aortic sinus, AAo, distal DAo, and infrarenal ABAo. The results indicated that age had a statistically significant positive correlation with measurements of the aortic diameter at the four above-mentioned levels—aortic sinus with *r* -value 0.275 ( *p* = 0.001 [95% CI = 0.193; 0.353]), for AAo the *r* -value was 0.580 ( *p* = 0.001 [95% CI = 0.519; 0.634]), for the distal DAo the *r* -value of 0.690 ( *p* = 0.001 [95% CI = 0.642; 0.733]), and for the infrarenal ABAo, *r* = 0.571 ( *p* = 0.001 [95% CI = 0.509; 0.626]). Similarly, the length of the AAo, the body mass index (BMI) of an individual, and SBP and DBP also demonstrated a statistically significant positive correlation to the diameter of the aorta at the above 4 referenced points ( Table 5 ).

**Table 5:** Correlation and association of risk factors with diameter of the aorta

Factors	Aortic sinus	Ascending aorta	Distal descending aorta	Infrarenal aorta
Age	*r* = 0.275 [95% CI = 0.193; 0.353]	*r* = 0.580 [95% CI = 0.519; 0.634]	*r* = 0.690 [95% CI = 0.641; 0.732]	*r* = 0.571 [95% CI = 0.509; 0.626]
Ascending aorta length	*r* = 0.487 [95% CI = 0.417; 0.550]	*r* = 0.420 [95% CI = 0.345; 0.488]	*r* = 0.536 [95% CI = 0.471; 0.595]	*r* = 0.476, [95% CI = 0.406; 0.540]
BMI	*r* = 0.156 [95% CI = 0.049; 0.258]	*r* = 0.204 [95% CI = 0.100; 0.304]	*r* = 0.236 [95% CI = 0.133; 0.334]	*r* = 0.215 [95% CI = 0.110; 0.314]
SBP	*r* = 0.094 [95% CI = 0.007; 0.180]	*r* = 0.239 [95% CI = 0.155; 0.320]	*r* = 0.326 [95% CI = 0.246; 0.402]	*r* = 0.276 [95% CI = 0.193; 0.354]
DBP	*r* = 0.107 [95% CI = 0.019; 0.192]	*r* = 0.172 [95% CI = 0.086; 0.255]	*r* = 0.272 [95% CI = 0.189; 0.351]	*r* = 0.218 [95% CI = 0.133; 0.299]

Abbreviations: BMI, body mass index; CI, confidence interval; DBP, diastolic blood pressure; SBP, systolic blood pressure.

## Discussion

This study evaluates the size of the aorta among a South Asian population with an endeavor to establish normal reference values. The significance of such population-based studies lies in the fact that in clinical practice, aortic dimensions have been used as the primary determinants, and there are only scarce studies defining the normal size of the aorta, 1 2 12 especially for geographical and ethnic subgroups.

The normal aorta is known to taper being the largest at the sinuses, and gradually decreasing in size over the ascending, arch, DAo, and thoracoabdominal aorta. 5 8 16 Our evaluation of the diameter of the aorta was analogous to that expectation, as the aortic diameter tapered down to the smallest diameter at the infrarenal segment of the aorta, similarly for both men and women.

The recent EACTS/STS (European Association for Cardio-Thoracic Surgery/Society of Thoracic Surgeons) guidelines have placed importance on patient-specific factors such as genetic variation, age, sex, family history, and body surface area (BSA) that should be taken into consideration while determining the diameters of the aorta and, hence, indication for intervention. 2 This study has reported on the dimensions of the aorta based on age, sex, and BSA ( Tables 2 – 3 ).

The size of the aorta is known to increase progressively with age. 1 2 4 5 6 7 8 9 17 In this study as well, age was found to have a positive correlation with the dimensions of the aorta at the level of the sinus, AAo, DAo, and infrarenal ABAo ( Table 5 ).

We assessed the aortic dimensions based on sex, finding that males do have a larger diameter aorta at most of the aortic reference sites, but this finding was not statistically significant, divergent to some previous studies 4 5 8 18 ( Tables 2 – 3 ). We also looked for an association of the diameter of the aorta with the BSA of an individual but contrary to studies and recommendations, 1 2 we did not find a significant association at any of the four measured sites (aortic sinus: *R* = 0.06, *p* = 0.26; AAo: *R* = (−)0.007, *p* = 0.89; DAo: *R* = 0.06, *p* = 0.179, and infrarenal aorta: *R* = 0.08, *p* = 0.096), nor did we find a statistically significant association with patient height. 1 2 However, we found a weakly positive correlation between the BMI of the individual and the aortic diameter at all the four reference points, similar to a study conducted by Obel et al. 5 ( Table 5 ).

While assessing for racial variation in dimensions of the aorta (as previously cited in literature), our study shows that the dimensions of the AAo, arch, and proximal DAo were marginally smaller than in Caucasians, but markedly smaller at the distal DAo and beyond 2 6 8 9 19 20 ( Table 3 ). While comparing the size of the AAo with studies comprising Caucasians and Asians, we found that the AAo was 33 ± 4 mm in Caucasians versus 28.9 ± 4.6 mm in a Korean population versus 29.3 ± 4.5 mm in our study. 6 10 Studies assessing the diameter of the AAo among Asians from the Korean Peninsula and China also determined that the AAo are smaller compared with Caucasians. These studies concluded that Asians, in general, had smaller AAo compared with the Western population. 10 20 Contrary to the above, the Multi-Ethnic Study of Atherosclerosis (MESA) study found that Chinese Americans had a larger-sized AAo in comparison to Caucasians, whereas Hispanics and African Americans had the smallest size AAo—however, the number of patients of Asian ethnicity assessed was too small to come to a definitive conclusion. But, more importantly MESA showed that aortic diameter variation exists among the races or ethnicity. 4

We compared the diameter of the aorta from our study to one conducted by Pham et al., who measured the dimensions of the aorta at various reference points in a population of Caucasians. We found that the size of the distal DAo, suprarenal, and infrarenal ABAo was larger than our population. 10 We found that the distal DAo in our population was 20.3 ± 3 mm versus 25 ± 2 mm; suprarenal ABAo was 19.5 ± 2.8mm versus 23 ± 2mm, and infrarenal ABAo was 15.5 ± 2.1 mm versus 20 ± 2 mm for males and 19.4 ± 2.9 mm versus 22 ± 2 mm, 18.8 ± 2.9 mm versus 21 ± 2 mm, and 14.7 ± 2.1 mm versus 17 ± 1.5 mm in females, respectively. 8 ABAo size in the Asian population has been previously reported to be small, with the infrarenal ABAo measured to be 1.6 to 1.8 cm in diameter. 21 22 One of the studies attributed the smaller stature of the Asian population to be the contributing factor. 21 We found that the size of the infrarenal ABAo was 15.3 ± 2.2mm in diameter and the size index of the infrarenal ABAo was 9.0 ± 1.3 mm in our population. We infer that the infrarenal ABAo is considerably much smaller in diameter even based on the size index among our population when compared with Caucasians. 4 10 23 Zafar et al., 9 while evaluating the natural history of DAo and thoracoabdominal aorta, concluded that almost 80% (94 of 118) patients suffered descending dissection below the size of 5cm. A similar review of the postmortem examination of fatal dissection cases concluded that the median size of the aorta was smaller than the current guideline threshold for elective repair. 24 Thus, based on our findings of the dimensions of the distal DAo and ABAo, we conclude that ruptures of the DAo and ABAO occur at much smaller sizes when compared with the AAo. 2 9 11 12 13 14 This is probably because the aorta at these sites is inherently smaller in diameter in some races or ethnic communities, as seen in our study. Thus, it becomes imperative that we investigate further the natural size of the aorta and its variation among different races and ethnicities. Such data will impact the indication for intervention for the DAo and ABAo.

Our study assessed the size of the aorta based on the height index as well, which is considered by some to be a more representative. Intervention is advised beyond 3.21. 9 25 We found that the size of the aorta based on height index was small, more so for the ABAo, with the suprarenal ABAo height index of 11.3 ± 1.8 mm and infrarenal ABAo of 9.0 ± 1.3 mm ( Table 4 ).

We assessed for factors that might affect the diameter of the aorta. We found that the length of the AAo has a significant positive relationship to the diameters of the aorta ( Table 5 ). The length of the AAo has been well recognized as clinically important and utilized in the past for risk stratification. The aorta is known to elongate as it dilates. 1 2 17 The recent EACTS/STS guidelines recommend considering an AAo length greater than 11 cm as a significant indicator to consider intervention. 2 In this study, the patients' SBP and DBP affected the diameter of the aorta with a positive association at all four reference points ( Table 5 ). Hypertension is known to be a risk factor for aortic dilatation, 5 9 17 20 with both SBP and DBP being related variables, consistently demonstrating a positive association with diameters of the ascending and DAo. 18 We did look for other influential and confounding factors for aortic size, such as sex, height of an individual, BSA, bovine arch, smoking, diabetes, hypertension, and dyslipidemia; none of which were found to have a statistically significant association in this study.

### Limitations

In this study, we used noncontrast and nonelectrocardiogram (non-ECG)-gated CT images of trauma patients who we believe would be more representative of individuals without disease or risk factors that could influence the size of the aorta. However, due to this method of assessment, we had to exclude 135 (19.2%) of the images due to motion artifacts that hindered accurate assessment of the dimensions. ECG-triggered or fast acquisition techniques are applied for minimizing motion artifacts, 2 and we recognize the importance of such techniques to acquire CT images, but indications for such imaging of the general population are extremely constrained. Further, although we have defined our measurement techniques for the aorta, there seems to be lack of consistent measurement techniques across literature.

Second, because our images were obtained from trauma patients predominantly of young ages, our findings may not completely represent the general population distribution in the community. However, we have addressed this by ascertaining aortic diameters based on age groups and their BSA.

## Conclusion

This study depicts the size of the aorta across reference points and shows that the normal size of the aorta, particularly the distal DAo and ABAo, is smaller in a South Asian population, even when indexed to BSA. Thus, racial or ethnic variation may play a significant role with regard to the dimensions of the aorta. The diameter of the aorta is affected by the age and the length of the AAo, the BMI of an individual, and SBP and DBP.
